# A portable geometry-independent tomographic system for gamma-ray, a next generation of nuclear waste characterization

**DOI:** 10.1038/s41598-023-39405-x

**Published:** 2023-07-28

**Authors:** Salvador Tortajada, Francisco Albiol, Luis Caballero, Alberto Albiol, José Luis Leganés-Nieto

**Affiliations:** 1 Instituto de Física Corpuscular, CSIC-Universitat de València, E-46980 Paterna València, Spain; 2 iTeam, Universitat Politècnica de València, E-46022 València, Spain; 3 ENRESA, Empresa Nacional de Residuos Radiactivos, Ingeniería de Residuos de Baja y Media Actividad, IRBMA, 28043 Madrid, Spain

**Keywords:** Nuclear energy, Nuclear waste

## Abstract

One of the main activities of the nuclear industry is the characterisation of radioactive waste based on the detection of gamma radiation. Large volumes of radioactive waste are classified according to their average activity, but often the radioactivity exceeds the maximum allowed by regulators in specific parts of the bulk. In addition, the detection of the radiation is currently based on static detection systems where the geometry of the bulk is fixed and well known. Furthermore, these systems are not portable and depend on the transport of waste to the places where the detection systems are located. However, there are situations where the geometry varies and where moving waste is complex. This is especially true in compromised situations.We present a new model for nuclear waste management based on a portable and geometry-independent tomographic system for three-dimensional image reconstruction for gamma radiation detection. The system relies on a combination of a gamma radiation camera and a visible camera that allows to visualise radioactivity using augmented reality and artificial computer vision techniques. This novel tomographic system has the potential to be a disruptive innovation in the nuclear industry for nuclear waste management.

## Introduction

The characterization of radioactive waste in nuclear power plants, both in operation and especially in decommissioning, is one of the main activities of the nuclear industry, which makes it possible to establish the classification of the waste and to define its final destination according to the existing management routes. The characterization and classification of radioactive nuclear waste, as well as the disposal of radioactive substances resulting from accidental contamination, is usually based on the detection of gamma radiation. The increasing requirements in regulations makes these processes costly and time-consuming, irrespective of whether portable or static detection systems are used, especially if they have to be carried out manually.

During nuclear waste management, large volumes of radioactive waste are often classified according to their average activity by volume or mass, where it is assumed that the radioactivity is homogeneously distributed. However, there are often situations where activity is inhomogeneously distributed. This arises to avoid that the maximum activity allowed by the regulator can be locally exceeded while the total activity in the volume is still under the margins. This situation should impose actions to redistribute or rearrange the material using different strategies that aim at time reduction, optimising decommissioning resources and budget or minimising radioactive exposure of operators.

A waste bulk from nuclear power plants often contains a set of major isotopes where the ratio between them changes depending on the system being treated^1,2^. The beta-gamma emitting radioisotopes whose photon energy is able to pass through the bulk can be measured from outside without the need for destructive testing. Conventional radiometers or dosimeters with no spectrometric capability are often used for these radioisotopes. However, it is possible to determine the gamma activity of these radioisotopes if the proportion of these radioisotopes are known a priori^3–5^. To a lesser extent, nuclear power plants have whole-bulk gamma spectrometer equipment. It is necessary for each bulk to be stored to determine the activity of both pure beta-emitting and alpha-emitting radioisotopes, which do not have sufficiently intense gamma-emitting energies to be detected from outside using non destructive methods. For these non-gamma-emitting radioisotopes, Scaling Factors (SF) have been developed. SF are based on an empirical methodology of correlation between gamma-emitting radioisotopes and non-gamma-emitting radioisotopes, where activation products are expected to correlate with the key radioisotope $^{60}\text {Co}$, while fission products are expected to correlate well with $^{137}\text {Cs}$^6^. Once the content of the key isotopes is known for each bulk, the SF of the rest of the beta and alpha emitters are applied, thus obtaining the activity of the complete set of radioisotopes present in the bulk. Hence, if the intensity and location of gamma-emitting radioisotopes can be detected, then a correlation with other non-gamma-emitting radioisotopes can be inferred.

For this purpose, we have developed a portable tomographic system for three-dimensional image reconstruction from gamma radiation detection. The system relies on the combination of a gamma camera^7^ with artificial computer vision, both providing a large Field-Of-View (FOV) that can identify, locate and quantify gamma-emitting radioisotopes in real time. This tomographic system is independent of the geometry of the radioactive source container as well as its position relative to the source term. Visible RGB cameras and fiducial markers are used to establish the coordinates of the system, allowing to retrieve the three-dimensional geometry of the scene. Unlike static systems that only provide measurements once the material is packaged, this system takes advantage of its small size and mobility to be deployed at the location where the staff still have access to the bulk.

## Results

The first result for the volumetric reconstruction is the convergence of the values of each voxel measured by means of the Average Euclidean Distance (AED).

**Figure 1:**
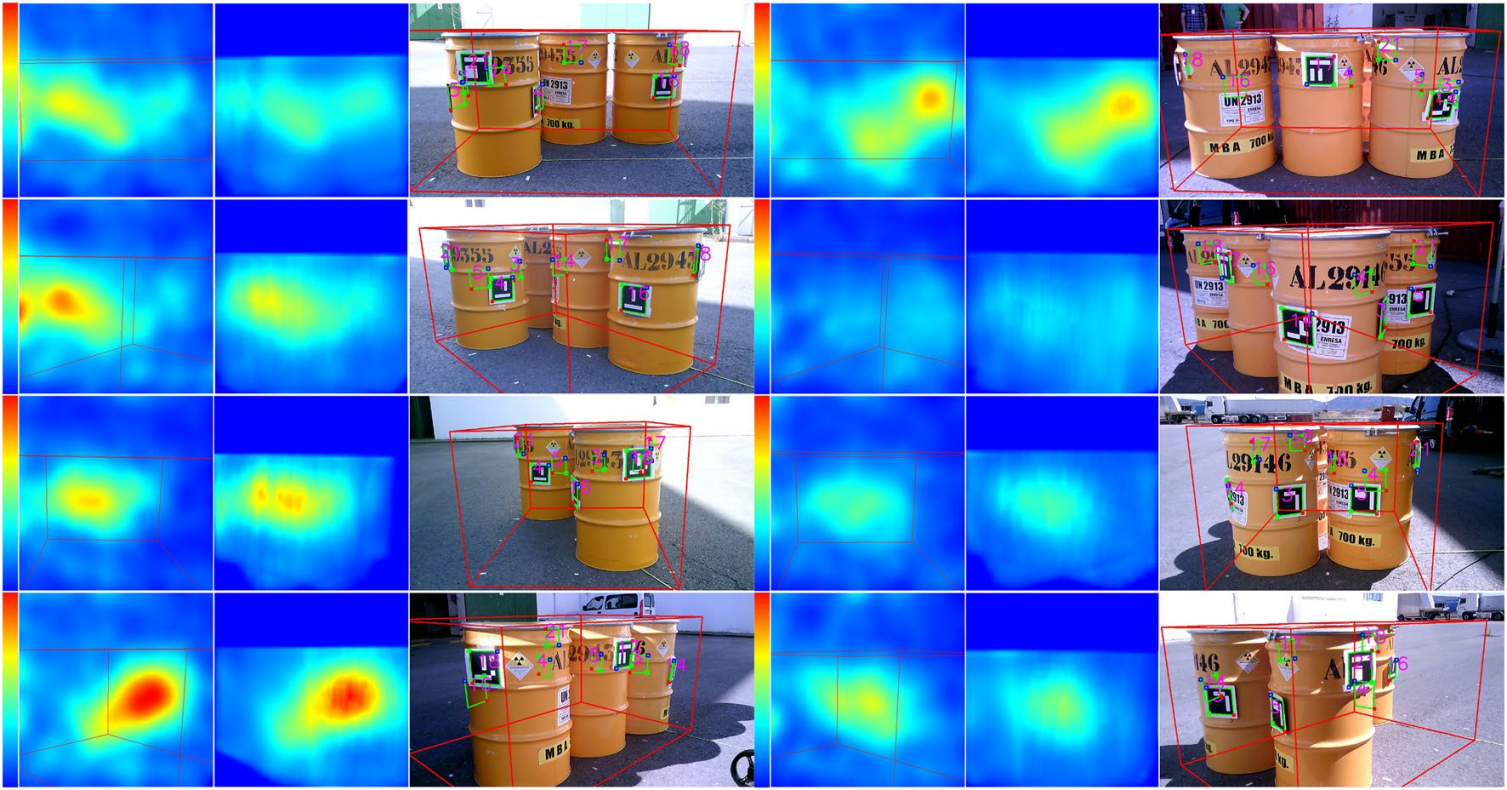
An example of the convergence of the algorithm for eight different acquisitions from eight different poses. For each pose, the figure shows the true gamma activity acquired image (left), the algorithm results after convergence (center) and the visible acquired image (right). The region of interest is delimited by the volume box (in red). The activity outside this region is removed for the reconstruction. The maximum activity per pixel is 40 KBq (red color) and the minimum activity corresponds to 0 Bq.

The results for the reconstructed planar gamma images also show a convergence from the initial iteration to the last iteration where the images of the gamma camera are reasonably well adjusted. This convergence is illustrated in Fig. 1. It is important to consider the parallax: slight changes in the apparent position of the objects are caused by the different viewpoints of each camera.

**Figure 2:**
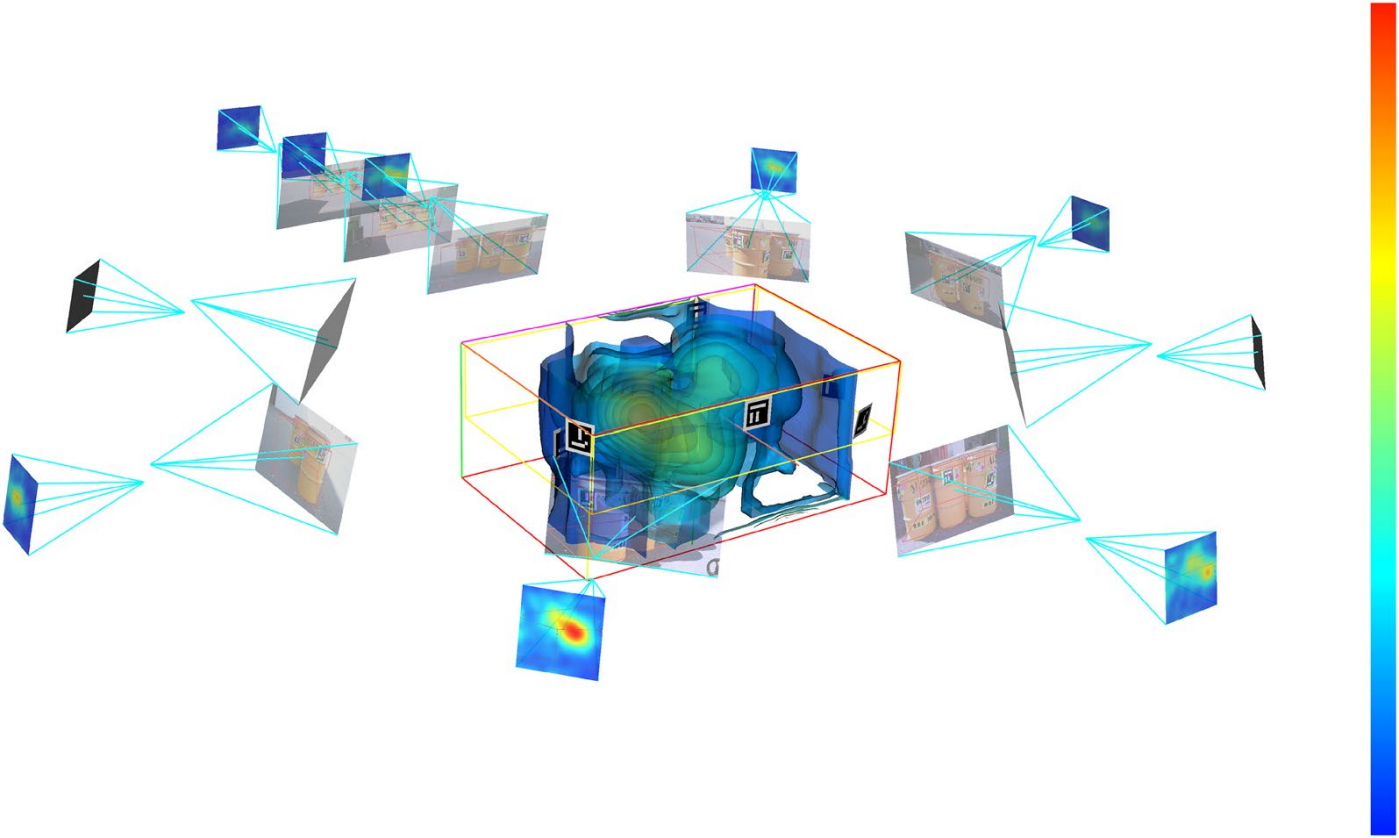
Example of the volumetric reconstruction of the radioactivity distribution of an acquired scene. The setting of the different camera poses enable the 3D reconstruction by stitching together the fiducial marker information. In addition, a magnifying glass effect can be done if the device is moved backwards following a straight line. The maximum activity per voxel is 487 KBq (red color), which corresponds to a maximum activity of 15, 7 KBq/cm$^3$.

For the 3D volumetric reconstruction of the gamma activity distribution, Fig. 2 shows an example of the projections that serve as a correct verification of the developed process. The figure shows the volumetric reconstruction of the complete scene. Since we retrieved the world coordinate system, it is possible to place the pose of each gamma and visible camera in the scene. Hence, it is feasible to see the projected gamma image of every single camera with the reconstruction of the activity lines of that camera; also, the image of each visible camera and their relative poses, the position of each fiducial marker and the volume box used for visual inspections and where the region of interest is located. The reconstructed tomographic activity distribution is shown inside this region.

For the drums included in the configuration shown in Fig. 6, each individual activity was measured following the current standardized protocol, i.e., from their contact dose rate, taking into account the nature of the waste, estimated density and the percentage composition of each gamma isotope contained in each drum. With this information, Activity-Dose conversion factors were calculated for each isotope contained. The radioisotopes were identified by gamma spectrometry and the total activity was assigned to each radioisotope according to the estimated percentages in the residue composition. Table 1 shows these values for the $^{60}$Co radioisotope. As can be shown with the method presented here, this information is valuably complemented with the results obtained from the tomographic activity distribution. It is important to note that the 3D reconstruction allows to complement the quantitative information to ensure that the drum content is homogeneously distributed in terms of the radioisotopes activity, avoiding heterogeneous distributions in which radioactive waste is concentrated in hot spots, thus improving and helping in decision-making for waste management. As expected, the activity quantified by the proposed method is nevertheless within the limits set by the current standardized protocol measurements.

**Table 1:**
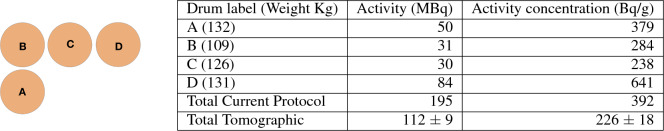
Activity measured for each drum following current protocols and total activity compared to the activity measured by the gamma camera detector. An illustration of the drum setting is shown on the left.

## Discussion

In the process of decommissioning a nuclear power plant, waste characterisation is a fundamental and mandatory process. A technology has been presented that combines a gamma and a visible camera to allow the recognition of the environment and reconstruction of the internal activity of a bulk with radioactive waste. Regardless of its complexity, the virtual segmentation of the source term into pieces of simple geometry that can be easily parameterised has proved to be a highly operative process that allows *in situ* measurements to be performed in a fairly agile manner and whose final result has proved to be highly robust and comparable to other more complex and less versatile processes.

Its use allows for the application of easily implemented processes in field that *a priori* seemed excessively complex to introduce in nuclear or radioactive facility decommissioning projects. Hence, it allows the detailed characterisation of radioactive wastes without slowing down the rest of the tasks associated with decommissioning.

The methods previously described are essential for the recovery and final projection of the radioactivity distribution of the nuclear waste being analysed. As explained in section Methods, the reconstruction process is not straightforward but it can achieve a precise tomographic reconstruction that is independent of any known geometrical setting. It must be considered that the activity detected in a pixel or region of pixels of the gamma camera integrates the activity of the areas crossed by the imaginary line. With the contribution of the different acquisitions, each voxel of the optimal volume can be reconstructed like a tomography. For each voxel, the contribution of each ray must be calculated, considering important factors such as attenuation, distance, intensity at the origin and total path travelled. These iterations have a high computational cost, as the dimensions in X, Y, and Z, the number of cameras and the dimensions of the beam must be considered.

In contrast to tomography systems dedicated to medical imaging such as the Single Photon Emission Computed Tomography (SPECT) imaging technique, where the geometry of the detectors is well-known, the gamma radiation tomograph presented here is independent of a spatial geometry that is unknown *a priori*. However, geometrical information is still of vital importance for the reconstruction of the radioactivity, so a methodology based on visible cameras and recognisable fiducial markers has been developed to recognise the environment and establish the reference system as described above.

The aim of our approach is to provide new, more versatile and user-friendly tools that provide useful and efficient three-dimensional gamma activity information to characterise nuclear waste that must meet certain requirements imposed by industry regulators. Design and development decisions are oriented to meet these requirements.

There are other approaches in the literature that can complement some of the aspects discussed here. Interesting contributions to the combination of visual and gamma-ray cameras are carried out elsewhere^8–10^. These devices have been developed that integrate 3D-LiDAR or MS Kinect imaging with gamma-ray detection (a topic called simultaneous localisation and mapping (SLAM) technology). These SLAM devices provide the ability to move freely to build a 3D model of the experimental environment and detect gamma radiation using Compton cameras, generating a Scene-Data Fusion that allows the radiation to be projected into a 3D visual representation. In Sato’s work^11^, a similar application has been developed, combining pinhole and Compton gamma camera with 3D-LiDAR technology and optical cameras that are combined by structure-from-motion to reproduce a 3D model of the gamma radiation.

We think that less complexity means greater reliability for industrial applications. Reducing the complexity of components, including software and reconstruction processes, simplifies the operations and increases operators safety and reliability. Step-by-step measurements for long time measuring can be safest than providing video-frames, acquisition-frames and a large number of associated elements where the gain could be minimal for this specific purpose. In fact, this exercise has been performed but difficulties arise when providing a protocol for moving detectors and keeping reliability and safety issues into account.

Increasing complexity may increase risks, such as image convergence, temporal mismatches, lighting issues or problems in communication with devices. This poses a handicap for the reliability and reproducibility of correct measurements, which translates into high costs due to the high volume of material that a nuclear power plant represents per year.

It is worth noting that our work introduces a contribution for the 3D reconstruction of gamma activity using a tomographic algorithm that is independent of a prior known geometry. The next generation of technologies for nuclear waste characterisation should include ways to improve the registration of images of different nature, to optimise real-time coordinate systems, to improve dose detection or to optimise the support systems of these technologies. In this respect, other developments point in similar directions for achieving some of these goals^8–11^.

We believe that our proposal can be extended as standard practice in nuclear power plants and other related potentially risk management activities, both in operation and in decommissioning. Hence, this novel tomographic system has the potential to be a disruptive innovation in the nuclear industry for nuclear waste management.

## Methods

### Gamma and visible cameras

The gamma detector used in this demonstrator consists of a pinhole gamma camera with an optical RGB camera attached to complement the gamma image information. The gamma camera is comprised of a gamma-ray detector that makes use of a LaBr$_3$ scintillation crystal of $51\times 51\times 15$ mm$^3$. The scintillation crystal is optically coupled to a Hamamatsu H10966A1DO Position-Sensitive Photomultiplier Tube (PSPMT) that reads 64 inputs and outputs up to 8 analogue signals which, by means of an integrated circuit AMIC2GR, allows to calculate the gamma-ray interaction position in the scintillation crystal. This planar gamma camera uses a lead pinhole collimator to avoid any gamma radiation not directly coming through its aperture^7,12^. These features allow the gamma camera to reconstruct the gamma image. It should be noted that the system has an internal self-calibration algorithm that takes advantage of the own activity of the scintillation crystals, due to the $^{138}$La decay^13^, so that it is not necessary to perform channel to energy calibrations with external sources throughout the measurement sessions, facilitating both the logistics and the dynamics of the measurements.

**Figure 3:**
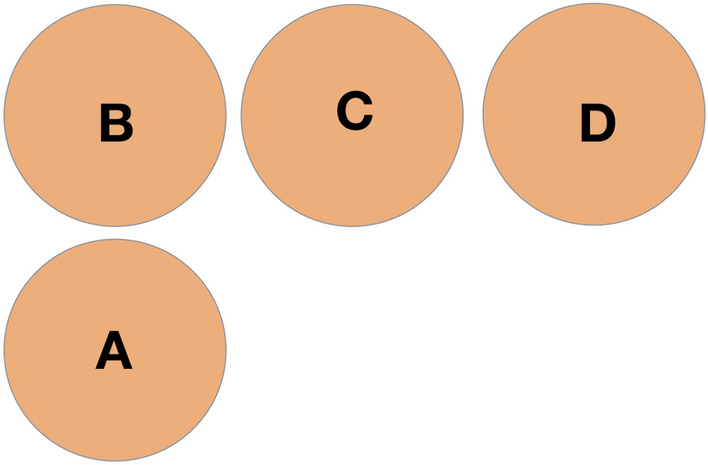
Illustration of the drum setting.

The RGB camera is a commercial Logitech C270 with $1280\times 720$ pixels. It is mounted over the gamma camera oriented roughly parallel to it to facilitate the estimation of the homography and provide easy rigid transformations. The RGB camera enables the application of computer vision techniques that make it possible to recognise the environment and accurately establish homographies between the different reference systems: world, gamma camera, and RGB camera. This setting enables the transformation of any 3D point into a planar 2D point and viceversa, while allowing the transformation of the so-called “gamma points” into “visible points”. These characteristics therefore allow the basis for a portable gamma tomography that is independent of the geometry of the scene (see Fig. 3). Any commercial camera with similar characteristics may be used. Commercial cameras are easy to purchase, calibrate, maintain and replace, and provide enough image quality. The choice is based over other more expensive tools in terms of reliability, providing enough precision for this purpose, and because minimal user intervention other than joint camera calibration is required^14^.

### Fiducial markers

The use of visible fiducial markers with known 3D coordinates is a helpful tool to determine extrinsic camera parameters and finding the camera pose from the images, given the marker reference system, with high speed and accuracy. There are many fiducial marker systems available, see for instance ARTag^15^, BinARyID^16^, Cantag^17^, Intersense^18^, or ReacTIVision^19^. Aruco marker framework^20^ was chosen because it provides methods for determining the location and orientation of a camera (the pose), based on the four corners of the square marker. Although the camera pose can be determined with just one fiducial marker with enough precision, we used a setting where many Aruco markers are detected. This allows the camera pose to be retrieved with higher precision and with robustness to possible occlusion of any marker. Aruco markers present also robustness against noise and vertex jitter. Additionally, Aruco has an available C++ library and utilities that allow a straight and fast integration in the system workflow.

### Geometric calibration

The goal of camera calibration is to recover the geometrical information from gamma-ray snapshots of the same volume containing radioisotopes generated from different angles and positions. The geometric calibration is based on the inference of projection matrices that map 3D points onto the 2D projected images. However, this process requires considering the high spatial variability of a detector that can move freely in the field. Hence, it is important to keep track of the different positions, distances and orientations of the detector with respect to the volume of interest if we intend to reconstruct the distribution of the internal radioactivity.

Since both cameras can be modelled as pinhole cameras, it is possible to apply projective geometry techniques^14,21^. To do this, it is mandatory to associate the projection matrices of each camera with a three-dimensional reference coordinate system. We also need to associate both cameras with each other by means of a rotation matrix and a translation vector that are calculated in this calibration stage.

In the pinhole camera model, the camera is modeled using a matrix camera $\textbf{P}$. This matrix consists of an intrinsic camera matrix, also called the *intrinsics* $\textbf{K}$, and an extrinsic camera matrix, or the *extrinsics*, that are composed by a rotation matrix $\textbf{R}$ and a translation vector $\textbf{t}$ with respect to the world coordinate system:

1 $$\begin{aligned} \textbf{P} = \textbf{K} \cdot [\textbf{R}|\textbf{t}] \end{aligned}$$

Hence, the projection of a 3D point $\textbf{X}$ in the world coordinate system as a 2D homogeneous point $\textbf{x}$ onto the image plane can be computed using the matrix camera:

2 $$\begin{aligned} \textbf{x} = \textbf{K} [\textbf{R}|\textbf{t}] \textbf{X} \end{aligned}$$

#### Intrinsic and extrinsic parameters

The intrinsic parameters $\textbf{K}$ can be decomposed as:

3 $$\begin{aligned} \textbf{K} = \begin{pmatrix}\alpha _x &{} 0 &{} x_0\\ 0 &{} \alpha _y &{} y_0\\ 0 &{} 0 &{} 1\end{pmatrix} = \begin{pmatrix}\lambda _x &{} 0 &{} 0\\ 0 &{} \lambda _y &{} 0\\ 0 &{} 0 &{} 1\end{pmatrix}\cdot \begin{pmatrix}f &{} 0 &{} p_x\\ 0 &{} f &{} p_y\\ 0 &{} 0 &{} 1\end{pmatrix} \end{aligned}$$

where $p_x, p_y$ are the coordinates of the principal point and *f* is the focal length. These components are expressed in *pixels*, but we can convert the points to spatial dimensions if we multiply it by a known camera resolution $\lambda$. It is conventionally assumed that $\lambda$ is independent of the axis, hence $\lambda _x = \lambda _y$.

In order to calibrate the intrinsic parameters of any camera, we need a set of 3D point to 2D point correspondences $\{ \textbf{X}_i \leftrightarrow \textbf{x}_i\}$. For any RGB camera, this set of point correspondences can be obtained using the well-known, fast, efficient, and straightforward Zhang’s calibration algorithm^22^ that is based on the detection of points on a checkerboard that is moved in different positions to get enough point correspondences.

The acquisition of a set of corresponding points for the calibration of the intrinsics of the gamma camera was carried out using a single radioactive $^{22}\text {Na}$ radioisotope source placed at different positions. We acquired 19 points whose 3D coordinates were known *a priori*. The 2D pixel coordinates of the gamma camera were obtained from the maximum value of the light distribution of the photocatode of the event position. Some corrections were made to avoid pin-cushion or border effects to get the *x*, *y*-pixel coordinates^7^.

We also considered a set of point correspondences between the gamma camera and the RGB camera to obtain the rigid transformation that relates the pixels from one camera to the pixels in the other camera. Hence, the corresponding points in the RGB camera were detected using a fiducial marker. Since the gamma camera has no lens present, we ignored spherical aberrations, radial or tangential distortions, and skew. Thus, we had 10 degrees of freedom at most: two focal distances, two principal points, three axes of rotation and three axes of translation. As each pair of corresponding points provides two linearly independent equations, at least 5 points of correspondence were needed. Hence, 19 points (38 available equations) were enough to accurately estimate the gamma camera matrix using the Direct Linear Transformation (DLT) algorithm^21^.

The extrinsic parameters represent a rigid transformation between different coordinate systems. This transformation is composed of the rotation and translation and is represented as $^\nu \textbf{T}_\omega = [\textbf{R}|\textbf{t}]$. Hence, $^\nu \textbf{T}_\omega$ and $^\gamma \textbf{T}_\omega$ are the transformations from the 3D world coordinate system ($\omega$) to the 3D coordinate system of the visible camera ($\nu$) and the gamma camera ($\gamma$), respectively.

In order to establish the rotation and translation between cameras we use the results of the calibrations of each camera where we obtained $^\nu \textbf{T}_\omega$ and $^\gamma \textbf{T}_\omega$. The rigid transformation between cameras can then be composed as:

4 $$\begin{aligned} ^\nu \textbf{T}_\gamma =\big ( ^\nu \textbf{T}_\omega \big ) \big (^\gamma \textbf{T}_\omega \big )^{-1} = \big ( ^\nu \textbf{T}_\omega \big ) \big (^\omega \textbf{T}_\gamma \big ) \end{aligned}$$

Hence, any point from the gamma camera ($\gamma$) coordinate system can be transformed to the RGB camera ($\nu$) coordinate system.

5 $$\begin{aligned} \textbf{X}_\nu =\big (^\nu \textbf{T}_\gamma \big ) \textbf{X}_\gamma \end{aligned}$$

### Data acquisition

Field work was carried out to acquire both gamma and visible images to evaluate the process of tomographic reconstruction of the bulk gamma activity distribution. The measurements were carried out at the *El Cabril* Disposal Facility. The working methodology was to carry out measurements with the equipment in different poses around a set of drums that had previously been placed and arranged in such a way that their configuration avoided distribution symmetries and also minimizing homogeneity in the activity between adjacent drums. In this way, both the capacity and the possible improvements of the developed algorithms can be analysed and checked with greater accuracy. Once the configuration of the containers was defined, the different fiducial markers were positioned on the surface of the drums, assuring that every measurement pose had always at least two markers on view, with the condition that there was always a common marker between contiguous poses. Acquisition times and distances were determined to ensure that the statistics were sufficiently relevant.

**Figure 4:**
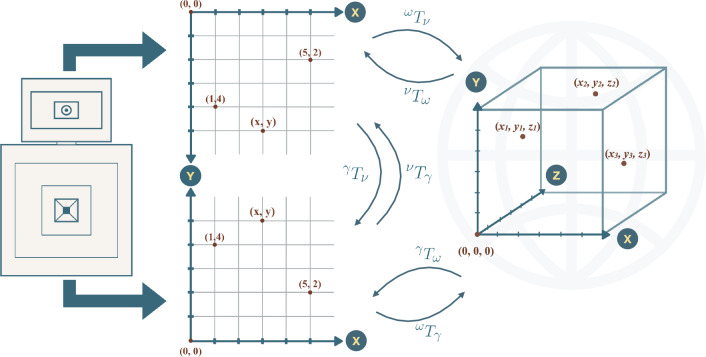
The upper device represents the visible camera. The bottom device represents the gamma camera. The different coordinate frames of each camera can move each point (pixel) to the other camera and to a 3D point (voxel) of the world coordinate system by means of the rigid transformations that have been calibrated.

The gamma-ray acquisition was made for the $^{60}$Co nuclide. The energy spectrum of the most active pose for this nuclide is shown in Fig. 4. the $^{60}$Co two photopeaks are identified and used for generating the radioisotope distribution image, as well as the peak of $^{40}$K from the concrete either in the drums and from the floor background and the region of the internal activity of the LaCl$_3$ scintillator. This is the specific spectrum used in these examples. Obviously, it is possible to do the detection and reconstruction on other energy spectra belonging to other isotopes.

### World reconstruction

The use of fiducial markers allows us to easily retrieve the camera pose with respect to the coordinate origin of the world reference system. This coordinate origin can be set at the user’s discretion or it can be arbitrary, in which case it will be the centre of the first fiducial marker detected.

The objective of camera pose estimation is to determine the three-dimensional position of a camera with respect to a known reference system. The complete 3D view of an acquisition can be reconstructed by stitching together the different fiducial markers found in a session or series of sessions. In addition, it is also possible to retrieve camera positions from this 3D view.

By recognising each of these fiducial markers, it is possible to identify the position of the marker, its rotation and translation vectors and the relative position of the markers to each other. This in turn allows to establish the relative positions of the RGB camera with respect to each set of markers detected in each visible image acquisition. Finally, thanks to the previously calibrated geometry between the RGB camera and the gamma camera, it is possible to identify the distribution of gamma activity to reconstruct later the radioactivity volume.

This environment recognition allows to get a first reconstruction of the gamma distribution by simply projecting the gamma images over a volume or region of interest based on the reference system retrieved by the RGB camera and the use of the fiducial markers.

### Algorithm for the reconstruction of the gamma distribution

The algorithm for the reconstruction of the volume distribution of gamma activity is based on similar algorithms that are applied in medical imaging tomographic systems for CT, PET and others such as those described in Refs.^23–27^. These algorithms are optimised assuming isocentric geometry distribution of the detectors, which is usually the arrangement found in commercial tomographic systems. As far as the location of the detector is manually placed by an operator, new algorithms have been developed to allow the reconstruction. The reconstruction of the volume is carried out using a forward-backward iterative optimization algorithm in which an initial reconstruction is projected with an arbitrary error and iteratively corrected to minimise an error function at each step until a stopping criterion is reached.


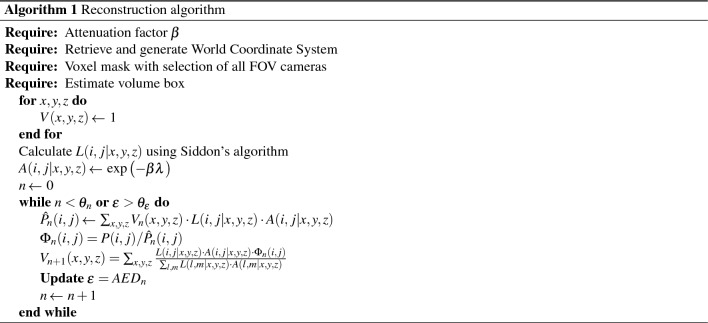


The algorithm initially requires an attenuation factor based on the weight of the bulk. Then, it retrieves and generate the world coordinate system as explained in subsection about the world reconstruction. Then, it selects a voxel mask and determines a volume box that contains the region of interest where the radioactivity is expected to be.

Then, the algorithm begins with an arbitrary initialisation of the value of each voxel of the acquired volume based on the planar images. This includes a classification to ensure that all pixels are under the FOV of all the detectors. After these requirements are met, the forward-backward optimization algorithm begins.

In the forward projection, the goal is to know the activity of the source term that is encompassed in each voxel *V*(*x*, *y*, *z*). This is estimated in an iterative way from an arbitrary initial value $V_0(x,y,z) = 1$. In this first step, the algorithm finds a correction factor for each pixel *P*(*i*, *j*) of the gamma image that allows to re-estimate the value of the voxel. For this purpose, the function to be calculated is the following:

6 $$\begin{aligned} {\hat{P}}_n(i,j) = \sum _{x,y,z} V_n(x,y,z) \cdot L(i,j|x,y,z) \cdot A(i,j|x,y,z) \end{aligned}$$

where ${\hat{P}}(i,j)$ is the estimated value for each pixel, $V_n(x,y,z)$ is the value of the voxel in the *n*-th iteration, *L*(*i*, *j*|*x*, *y*, *z*) is the length of the ray of pixel *P*(*i*, *j*) passing through voxel (*x*, *y*, *z*) and *A*(*i*, *j*|*x*, *y*, *z*) is the attenuation value of that same ray in voxel (*x*, *y*, *z*).

The ray of an (*i*, *j*)-th pixel will pass through a number of voxels and each of them will share a fraction of that ray. The length *L*(*i*, *j*|*x*, *y*, *z*) passing through voxel (*x*, *y*, *z*) from the ray of pixel (*i*, *j*) is the portion of that same ray that goes through that particular voxel. It is calculated using a modified Siddon’s algorithm for the exact path^28^. Siddon’s algorithm returns the point of entry and exit of each voxel for each ray that passes throughout that voxel and gives the length that is associated with value *L*(*i*, *j*|*x*, *y*, *z*), also called the radiological path.

The attenuation value *A*(*i*, *j*|*x*, *y*, *z*) is the value calculated from the time the camera pixel (*i*, *j*) beam enters the volume of the bins until it enters the voxel in (*x*, *y*, *z*). This attenuation is calculated as

7 $$\begin{aligned} A(i,j|x,y,z) = \exp \big ({-\beta \lambda }\big ) \end{aligned}$$

where $\beta$ is the inverse of the attenuation length at (*x*, *y*, *z*) and $\lambda$ is the length travelled by the ray in that voxel.

Subsequently, the algorithm corrects the voxel values iteratively using a correction factor $\Phi (i,j)$ that is calculated based on the actual pixel value *P*(*i*, *j*) and a pixel estimate ${\hat{P}}(i,j)$:

8 $$\begin{aligned} \Phi _n(i,j) = \frac{P(i,j)}{{\hat{P}}_n(i,j)} \end{aligned}$$

This allows to re-estimate the value of each voxel. These calculations are carried out at pixel level, but the calculation at the camera level has to be taken into account for the normalisation of the voxel value. This involves integrating the values for each pixel. Obviously, the integrals are not continuous but are discretised in order to be computed.

In the backward projection, the computation of the new voxel value for the next iteration $n+1$ is calculated at the end of iteration *n* according to the following normalised formula:

9 $$\begin{aligned} V_{n+1}(x,y,z) = \sum _{x,y,z}\frac{L(i,j|x,y,z)\cdot A(i,j|x,y,z)\cdot \Phi _n(i,j)}{\sum _{l,m}L(l,m|x,y,z)\cdot A(l,m|x,y,z)} \end{aligned}$$

The algorithm keeps adjusting the value of each voxel, which represents the estimate of the accumulation of source terms, until a stopping criterion is reached. The stopping criterion can be one of these three criteria or a combination of them: the number of iterations, the convergence of the values of voxels, or the convergence of an error function.

The stopping criterion for the convergence of the voxels of the volumetric reconstruction was measured using the AED for each iteration, which is equal to the root mean square deviation times $1/\sqrt{N}$:

10 $$\begin{aligned} AED_i = \frac{1}{N}\sqrt{\sum _{n=1}^N{\Big (V_n^{(i+1)}-V_n^{(i)}\Big )^2}} \end{aligned}$$

**Figure 5:**
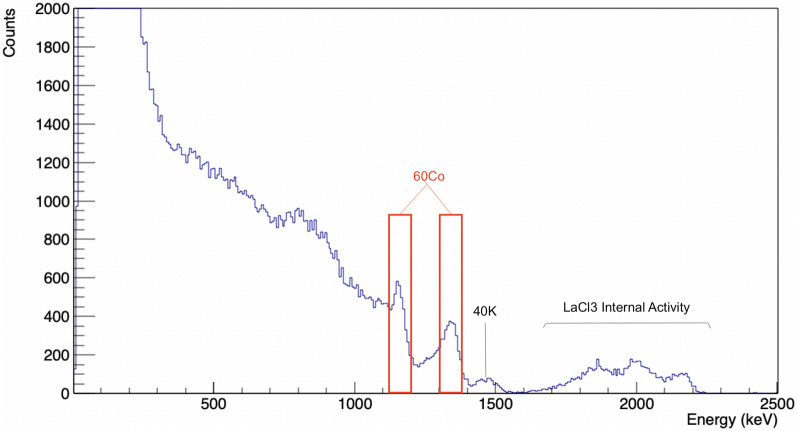
Measured energy spectrum of the most active pose in which the $^{60}$Co two photopeaks are identified and used for generating the radioisotope distribution image (gates marked in red), as well as the peak of $^{40}$K from the concrete either in the drums and from the floor background and the region of the internal activity of the LaCl$_3$ scintillator.

The initial discretisation was carried out at pixel-level. However, discretising the computation at pixel-level showed a significant aliasing problem. Aliasing was particularly noticeable in the areas of lower beam penetration density, i.e. the areas furthest away from the camera. The solution to this phenomenon was to carry out super-resampling, which reduces aliasing by artificially increasing the number of rays, modifying Siddon’s algorithm to take this information into account, hence improving the resolution. Figure 5 shows the aliasing problem on the left and the supersampling solution on the right.

### Radioisotope activity quantification and visualization

The whole reconstruction is based on gamma-ray activity quantification and its distribution. Each isotope has an associated efficiency matrix that allows us to recover the quantitative activity. Hence, we can consider the different gamma-ray energies for different nuclides. The efficiency matrices convert event counts at the space cell of the detector to pure activity at one meter. The total activity is measured in Bq. Although dose is also quantified in the nuclear industry in mSv. In addition, it is adjusted according to the pixel and voxel resolution respectively. For example, if the resolution is doubled, then the activity must be scaled to 1/4 in the case of planar image and to 1/8 in case of reconstructed volume. These calculations are considered when cameras need to oversample the resolution in the algorithm to avoid aliasing.

**Figure 6:**
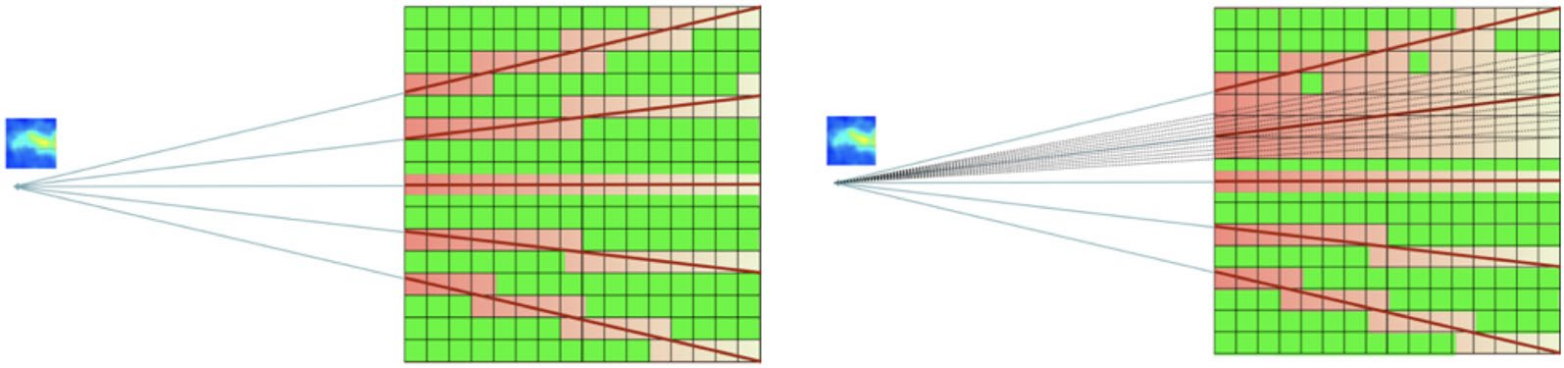
Illustration of the original sampling (left), where significant aliasing occurs because there are fewer rays. Application of super-resampling (right), where fractions of pixels are considered for higher ray sampling, which drastically reduces aliasing.

The quantitative activities are then used for bidimensional and tridimensional visualization and reconstruction. The maximum detected activity from all the different acquisitions and camera poses is used as the maximum value. This is assigned a $100\%$ value. Then, each of the acquisition corresponding to a pose of the complete set is shown adjusted to this common maximum. In this way, it is possible to observe the different activity intensities detected in each acquisition and compare them. This notation allows us to obtain color scales with the maximum dynamic range. If we do not normalize in this way the detected activity, we would lose the global maximum and the ability to assess which regions contribute more to it. The main advantage of expressing the activity in terms of percentage is that users can have information about how to rearrange the waste before closing the container, still having the total activity not to exceed the regulator recommendations. In the results section, total activity and maximum and minimum activity are shown for the acquisition of Figs. 1 and 6.

## Data Availability

Supplementary material, data availability, and videos generated and analysed during the development of this project are available at this GIT repository link.
